# Supplementary material for: LDA filter: A Latent Dirichlet Allocation preprocess method for Weka
Source: PLoS One. 2020 Nov 9;15(11):e0241701. doi: 10.1371/journal.pone.0241701 (PMC7652301; doi:10.1371/journal.pone.0241701)
Supplement: Attachment — Submitted filename: ResponseToReviewers.pdf [file pone.0241701.s001.pdf]

Title: “LDA Filter: A Latent Dirichlet Allocation preprocess method for Weka”
Authors: P. Celard, A. Seara Vieira, E. L. Iglesias and L. Borrajo

Dear Dr. Bonizzoni,

Thank you for giving us the opportunity to submit a revised draft of the manuscript “LDA Filter: A Latent Dirichlet Allocation preprocess method for Weka”. We appreciate the time and effort that you and the reviewer dedicated to providing feedback on our manuscript and are grateful for the insightful comments on and valuable improvements to our paper. We have incorporated most of the suggestions made by the reviewers. Please see below, in blue, for a point-by-point response to the reviewers’ comments and concerns.

The authors should particularly strive to address the following issues raised in review:

Can the authors propose an explanation for the fact that, for all datasets used, the LDA method systematically only improved the results of the kNN algorithm, but not SVM and NB?

Author response: Thank you for pointing this out. Dimension reduction negatively affects the SVM and NB classification algorithms, while the elimination of dimensions formed mostly by zeros in the BoW improves the k-NN algorithm. A wider explanation and new references that support this theory have been added. Please refer to the manuscript.

More data sets should be tested (possibly openly accessible ones as well), so the proposed LDA filter can be shown to provide significant improvements for a larger range of datasets.

Author response: As suggested by the reviewer, we have tested more data sets that support the originally obtained results. The new data sets are as follows.

- TREC Genomics 2005: It is a full-text document collection consisting of biomedical journal articles. We use one of the categories it is made of and classify the documents as relevant or not relevant.
- Yelp Polarity dataset: It is extracted from the Yelp Dataset Challenge 2015 data being a popular dataset for text classification and sentiment analysis. This is a biclass data set considering star rating 1 and 2 negative, and 3 and 4 positive.
- Yahoo! Answers dataset: Includes Yahoo! Research questions and their corresponding answers using the 10 largest main categories as classes.

All the previous datasets and two of those that were already included in the original paper (Reuters-21578 and 20 Newsgroup) can be accessed freely and openly on their respective websites. The only one that is not freely accessible is OHSUMED, but because we do not own it, we cannot distribute it.

A broader literature survey should help to contextualize the problem statement, and should inform a discussion of the reasons why LDA improves text classification performance.

Author response: We have added the suggested content to the manuscript on the introduction section. We have included new references that help to contextualize the use of LDA in the current landscape and the need to include this technique in a widely used tool like weka.

In addition to source code, the authors should also share compile and install instructions to ensure reproducibility of the work described here, as well as documentation for the use of their plugin.

Author response: Following your recommendations compile and install instructions have been added both to Github and Sourceforge. These instructions also include the steps to use the plugin in weka, from loading data to the last step of applying a classifier to the new text representation. Please refer to the user manual document, to Github README.md file or to Sourceforge main wiki page.

Journal Requirements:

When submitting your revision, we need you to address these additional requirements.

1. Please ensure that your manuscript meets PLOS ONE's style requirements, including those for file naming. The PLOS ONE style templates can be found at

https://journals.plos.org/plosone/s/file?id=wjVg/PLOSOne_formatting_sample_main_body.pdf and

https://journals.plos.org/plosone/s/file?id=ba62/PLOSOne_formatting_sample_title_authors_affiliations.pdf

Author response: As requested, the manuscript meets all the requirements in the style sheets.

2.We note that the figures in your submission contain copyrighted images. All PLOS content is published under the Creative Commons Attribution License (CC BY 4.0), which means that the manuscript, images, and Supporting Information files will be freely available online, and any third party is permitted to access, download, copy, distribute, and use these materials in any way, even commercially, with proper attribution. For more information, see our copyright guidelines: http://journals.plos.org/plosone/s/licenses-and-copyright.

Author response: The referenced image (Fig.1) has been replaced by a new figure to which the authors of this paper fully own the rights.

3.Thank you for stating the following in the Funding Section of your manuscript:

[This work was partially supported by the Consellera de Educacion, Universidades e Formacion Profesional (Xunta de Galicia) under the scope of the strategic funding of ED431C2018/55-GRC Competitive Reference Group.]

We note that you have provided funding information that is not currently declared in your Funding Statement. However, funding information should not appear in the Acknowledgments section or other areas of your manuscript. We will only publish funding information present in the Funding Statement section of the online submission form.

Please remove any funding-related text from the manuscript and let us know how you would like to update your Funding Statement. Currently, your Funding Statement reads as follows:

[The author(s) received no specific funding for this work.]

Author response: The Funding section of the manuscript has been removed. The funding statement does not need to be updated, remaining as [The authors did not receive specific funding for this work.]

Review Comments to the Author

Reviewer #1:

*** What are the main claims of the paper and how significant are they for the discipline?**

The main objective is to create a filter for Weka, where text data could be transformed in the low dimension representation using LDA and show that the classification tasks using LDA representation are faster without compromising accuracy.

Using LDA for text representation has been in practice for several years now. So, there is no new research contribution. The only contribution from the authors is the creation of an LDA plugin for Weka.

*** Are the claims properly placed in the context of the previous literature? Have the authors treated the literature fairly?**

No, there are a lot of papers in the literature that uses LDA for information retrieval, search engines, text matching, text hashing, etc. The authors have just cited the base paper by Blei and the semi-supervised extension on LDA modeling. The literature survey is insufficient in the context of the problem statement.

Author response: Thank you for pointing this out. The reviewer is correct, and we have added the suggested content to the manuscript on the introduction section. We have included new references that help to contextualize the use of LDA in the current landscape and the need to include this technique in a widely used tool like weka.

*** Do the data and analyses fully support the claims? If not, what other evidence is required?**

The results of the experiments are presented in the paper. The authors have used LDA (by calling an API from the MALLET library) to build a filter for Weka. As per their own experimental results, the filter appears to be not useful for improved classification accuracy. In all the 3 datasets used in the experiments, the LDA method worked for just the kNN algorithm, but no reasoning provided. Also, there is no explanation provided for why the method didn't work for other algorithms (SVM and NB). Using just 3 datasets for the experiments seem insufficient to prove anything empirically. The authors claim "speed" improvement as a positive outcome, but it is not interesting as with any dimensional reduction technique speed improvement is obvious.

Author response: We agree with the reviewer's assessment. Dimension reduction negatively affects the SVM and NB classification algorithms, while the elimination of dimensions formed mostly by zeros in the BoW improves the k-NN. Accordingly, throughout the manuscript, a wider explanation and new references that support this theory have been added.

*** If the paper is considered unsuitable for publication in its present form, does the study itself show sufficient potential that the authors should be encouraged to resubmit a revised version?**

Yes, creation of an LDA filter for Weka is an useful contribution, but the authors should improve the LDA method to make the filter help in improving the classification accuracy.

Author response: Thank you for your comments. The idea seems to us very interesting, so it will be taken into consideration in our research.

Some of the suggested amendments are:

+ More data sets to be tested.

Author response: As suggested by the reviewer, we have tested more data sets that support the originally obtained results. The new data sets are as follows.

TREC Genomics 2005: It is a full-text document collection consisting of biomedical journal articles. We use one of the categories it is made of and classify the documents as relevant or not relevant.

Yelp Polarity dataset: It is extracted from the Yelp Dataset Challenge 2015 data being a popular dataset for text classification and sentiment analysis. This is a biclass data set considering star rating 1 and 2 negative, and 3 and 4 positive.

Yahoo! Answers dataset: Includes Yahoo! Research questions and their corresponding answers using the 10 largest main categories as classes.

+ LDA filter should be shown providing accuracy improvement for the majority of the datasets.

Author response: As explained above, LDA offers improved results for all data sets using the k-NN algorithm and for multi-class data sets using the SVM classifier. Experimenting with new data sets, as suggested by the reviewer, supports these results.

+ Thorough literature survey should be done to find cues for how to LDA for improving text classification performance.

Author response: New references have been added. Please refer to the manuscript.

+ The LDA tuning process can become costly if a grid search for parameters is done. So, a method for smart tuning should be suggested.

Author response: We think this is an excellent suggestion and will be taken into consideration in future works.

+ Source code is made available, but the preprocesed dataset and results are not available in public domain. Sufficient documentation of the source code should be provided with compile and install instructions.

Author response: All datasets can be accessed freely and openly on their respective websites.

*** Are original data deposited in appropriate repositories and accession/version numbers provided for genes, proteins, mutants, diseases, etc.?**

No. Data is not made available. The source code is made available in Github, but there are no instructions for compilation and testing. There is no documentation available for how to tune/use the plugin. The plugin is made available in Sourceforge, but no documentation either.

Author response: Thank you for your pertinent suggestion. Compile and install instructions have been added both to Github and Sourceforge. These instructions also include the steps to use the plugin in weka, from loading data to the last step of applying a classifier to the new text representation. Please refer to Github README.md file and Sourceforge main wiki page.

*** Are details of the methodology sufficient to allow the experiments to be reproduced?**

Yes, if we the use the plugin prebuilt for Weka (https://sourceforge.net/projects/weka-lda-filter/)

If we just follow the paper, it is not possible to reproduce the experiment.

Author response: An additional document is included with the instructions for installing and using the LDA plugin, as well as links to download the data sets used in the experiments. These same instructions have been included in a README.md file on Github and a Wiki page on Sourceforge, places from which it can be downloaded.

*** Is the manuscript well organized and written clearly enough to be accessible to non-specialists?**

The paper is written like a technical report and not like a research article.

Author response: We agree with the reviewer's assessment. The most technical part of the paper has been removed and taken to a user instructions document. We have focused the paper on the proposal of the model and the review of the results achieved.
